# Transcription Factor TonEBP Stimulates Hyperosmolality-Dependent Arginine Vasopressin Gene Expression in the Mouse Hypothalamus

**DOI:** 10.3389/fendo.2021.627343

**Published:** 2021-03-16

**Authors:** Dong Hee Kim, Kwang Kon Kim, Tae Hwan Lee, Hyejin Eom, Jin Woo Kim, Jeong Woo Park, Jin Kwon Jeong, Byung Ju Lee

**Affiliations:** ^1^ Department of Biological Sciences, College of Natural Sciences, University of Ulsan, Ulsan, South Korea; ^2^ Department of Pharmacology and Physiology, School of Medicine & Health Sciences, The George Washington University, Washington, DC, United States

**Keywords:** TonEBP, hyperosmolality, arginine vasopressin, hypothalamus, appetite behavior

## Abstract

The hypothalamic neuroendocrine system is strongly implicated in body energy homeostasis. In particular, the degree of production and release of arginine vasopressin (AVP) in the hypothalamus is affected by plasma osmolality, and that hypothalamic AVP is responsible for thirst and osmolality-dependent water and metabolic balance. However, the osmolality-responsive intracellular mechanism within AVP cells that regulates AVP synthesis is not clearly understood. Here, we report a role for tonicity-responsive enhancer binding protein (TonEBP), a transcription factor sensitive to cellular tonicity, in regulating osmosensitive hypothalamic AVP gene transcription. Our immunohistochemical work shows that hypothalamic AVP cellular activity, as recognized by c-fos, was enhanced in parallel with an elevation in TonEBP expression within AVP cells following water deprivation. Interestingly, our *in vitro* investigations found a synchronized pattern of TonEBP and AVP gene expression in response to osmotic stress. Those results indicate a positive correlation between hypothalamic TonEBP and AVP production during dehydration. Promoter and chromatin immunoprecipitation assays confirmed that TonEBP can bind directly to conserved binding motifs in the 5’-flanking promoter regions of the AVP gene. Furthermore, dehydration- and TonEBP-mediated hypothalamic AVP gene activation was reduced in TonEBP haploinsufficiency mice, compared with wild TonEBP homozygote animals. Therefore, our result support the idea that TonEBP is directly necessary, at least in part, for the elevation of AVP transcription in dehydration conditions. Additionally, dehydration-induced reductions in body weight were rescued in TonEBP haploinsufficiency mice. Altogether, our results demonstrate an intracellular machinery within hypothalamic AVP cells that is responsible for dehydration-induced AVP synthesis.

## Introduction

The fluid and metabolic systems are reciprocally interconnected, and body fluid status, i.e., hydration or dehydration, strongly influences a broad spectrum of metabolic parameters, including food intake, weight gain, and energy expenditure, in both human and non-human species (1–5). Accumulated information points to the central nervous system (CNS) as a key site for crosstalk between the fluid and metabolic systems (6, 7). Within the CNS, the forebrain sensory circumventricular organs (CVOs), including the subfornical organ (SFO) and the organum vasculosum of the lamina terminalis (OVLT), form a complex molecular and cellular machinery that senses fluid information and plasma osmolality (8–12). Synaptic fibers from the SFO and OVLT project into the hypothalamic nuclei, such as the paraventricular (PVN) and supraoptic nuclei (SON) (8, 10, 13–16). Those hypothalamic nuclei have been recognized as the forebrain metabolic center; they integrate central and peripheral water and metabolic information and regulate neuroendocrine outputs, including the production and release of arginine vasopressin (AVP), in response to the body’s needs.

In the brain, AVP is synthesized predominantly in magnocellular and parvocellular neurons of the PVN and SON, and then it is translocated and stored in the neurohypophysis (17, 18). Traditionally, the hypothalamic AVP system has been highlighted in the regulation of water homeostasis (3, 10, 19–21). Dehydration causes an increase in blood osmolality as fluid volume is reduced, and that water-restricted condition activates the release of hypothalamic AVP to stimulate the kidneys for water reabsorption (19–21). On the other hand, SFO excitatory neurons predict the body’s need for fluid homeostasis following eating and drinking and activate the release of hypothalamic AVP to induce thirst-dependent drinking behavior in awake animals before any change in blood osmolality can occur (8, 10). Therefore, the hypothalamic AVP system plays a central role in both intero-sensory regulation and the rapid anticipatory drinking response that promotes fluid homeostasis (3).

In addition to water homeostasis, hypothalamic AVP is directly implicated in metabolism regulation. Alterations in the plasma AVP concentration are associated with the development of metabolic disorders (4, 5, 22–28). For example, AVP resistance, a chronic elevation in plasma AVP levels and decreased responsiveness of AVP receptors, is a leading factor in the development of obesity caused by increased food intake and impaired glucose metabolism (24). Overactivation of the AVP system is also common in insulin-dependent and -independent diabetic conditions (22, 23, 25) and has been linked with impaired lipid metabolism, insulin and glucose signaling, and energy expenditure (5, 26–28). On the other hand, acute activation of the hypothalamic AVP system in normal conditions increases the plasma AVP level and attenuates food intake (7).

As aforementioned, crosstalk between the water and metabolic systems occurs through the hypothalamic AVP; therefore, hypothalamic AVP is critical in the tight regulation of plasma AVP levels. To date, the mechanisms leading to the release of AVP have been well characterized (19, 21, 27, 29, 30). However, keeping in mind that plasma hyperosmolality is a powerful single stimulator for AVP production, the intracellular molecular mechanisms that activate AVP gene transcription within hypothalamic AVP cells in response to dehydration or increased plasma osmolality remain unclear.

Interestingly, a previous investigation in chickens suggested the possible involvement of tonicity-responsive enhancer binding protein (TonEBP) in hyperosmolality-dependent hypothalamic AVP gene expression (31). TonEBP is a ubiquitously distributed transcription factor that can bind to tonicity-responsive enhancer sites through a Rel-like DNA motif (32). TonEBP activity is stimulated by extracellular hypertonicity and activates the expression of downstream osmoprotective molecules in response to osmotic stress to support the survival and osmoadaptation of cells (32–36). Especially in rat brains, cells producing TonEBP have been detected in the PVN and SON, where AVP cell bodies are also densely distributed. The expression of TonEBP within those nuclei is upregulated by hypertonicity (37). However, no previous investigations have determined whether TonEBP and the AVP gene interact directly or indirectly upstream of dehydration-induced hypothalamic AVP gene transcription.

Therefore, we here investigated whether TonEBP is expressed in AVP neurons in the PVN and SON and whether it regulates AVP gene transcription in response to dehydration-induced hyperosmolality in rodents. Our results demonstrate that 1) expression of TonEBP in the PVN and SON is activated by hyperosmolality, 2) TonEBP can directly bind to the 5’ flanking promoter region of the AVP gene, 3) TonEBP stimulates hyperosmolality-dependent hypothalamic AVP gene transcription, and 4) the hypothalamic TonEBP-AVP system is associated with dehydration-induced reductions in body weight. Altogether, our results provide a molecular mechanism for the regulation of osmosensitive AVP gene transcription in hypothalamic AVP cells.

## Materials and Methods

### Animals

Eight-week-old C57BL/6 mice were used for animal experiments, which were conducted in accordance with the regulations of the University of Ulsan for the Care and Use of Laboratory Animals (permission number, BJL-20-050). Mice were housed in a room with a conditioned photoperiod (12-h light/dark cycle; lights on from 6:00 a.m. to 6:00 p.m.) and temperature (22–25°C) and allowed *ad libitum* access to tap water and standard pelleted mouse chow. To identify the *in vivo* effects of TonEBP on AVP mRNA expression in the hypothalamus, heterozygous TonEBP haploinsufficiency [TonEBP (+/-)] mice were used (a kind gift from Dr. H.M. Kwon, UNIST, Ulsan, South Korea). TonEBP knockout animals die during development, so TonEBP haploinsufficiency mice have been used extensively from us and other groups to study the physiological functions of TonEBP *in vivo* (38–44). To determine whether TonEBP affects dehydration-induced energy homeostasis, drinking water was withdrawn for 48 h, starting at 10:00 a.m., with food available *ad libitum*, and the food intake and body weight of wild and TonEBP haploinsufficiency mice were measured every day for 2 days.

### Immunohistochemistry (IHC)

Mice were anesthetized with tribromoethanol and transcardially perfused with phosphate buffer (PB, 0.1 M, pH 7.4), followed by a fresh fixative of 4% paraformaldehyde in PB. Brains were post-fixed overnight at 4°C, sliced to a thickness of 50 μm using a vibratome (VT1000P; Leica Microsystems, Wetzlar, Germany) and reference to the *Mouse Brain Atlas* (Paxinos G and Franklin KBJ, 2001, Academic Press, San Diego, CA, USA) to extract the PVN and SON, which were washed several times in PB. Coronal brain sections were pre-incubated with 0.2% Triton X-100 (T8787, Sigma-Aldrich, St. Louis, MO, USA) in PB for 30 min to permeabilize the tissues and cells. After further washing with PB, the sections were incubated overnight at room temperature (RT) with goat anti-TonEBP antibody (1:1000, Santa Cruz, Dallas, TX, USA, Catalogue No. sc-5501), mouse monoclonal anti-AVP antibody (1:1000, Santa Cruz, Catalogue No. sc-390723), and rabbit anti-c-Fos antibody (1:1000, Millipore, Billerica, MA, USA, Catalogue No. ABE457). The next day, the sections were washed in PB and incubated with the following secondary antibodies for 2 h at RT: Alexa Fluor 594-labeled anti-goat antibody, Alexa Fluor 594-labeled anti-rabbit antibody, or Alexa Fluor 488-labeled anti-mouse antibody (1:2000 each, Life Technologies, Carlsbad, CA, USA). Sections were mounted onto glass slides, and coverslips were placed and sealed with nail polish. Immunofluorescence images were captured using an FV1200 confocal laser-scanning microscope (Olympus America, Inc., Center Valley, PA, USA). The number of immunostained cells from both sides of the PVN and SON was measured manually by an unbiased observer using ImageJ software (National Institutes of Health, Bethesda, MD; http://rsbweb.nih.gov/ij/).

### Cell Culture and Transfection

We have utilized mHypoA 2/28 cell line in this study, as this cell line was originally generated from the hypothalamus, and therefore, includes a broad library of hypothalamic neuronal phenotypes (44, 45). mHypoA 2/28 cells (CELLutions Biosystems Inc. Ontario, Canada) were maintained in high-glucose Dulbecco’s Modified Eagle Medium (Hyclone, South Logan, UT, USA) supplemented with 10% fetal bovine serum and 100 U/ml penicillin-streptomycin (Hyclone) in a humidified atmosphere with 5% CO_2_ at 37°C. When the cells had grown to 70% confluence, they were transiently transfected with expression vectors or small interfering RNA (siRNA) using jetPRIME**^®^** reagent (Polyplus, New York, NY, USA): expression vector pCMV-myc containing the TonEBP coding region (a gift from Dr. H.M. Kwon, 500 ng) or control pCMV-myc; TonEBP siRNA (50 nM, sense sequence, 5’-GAC CAU GGU CCA AAU GCA A-3’; antisense sequence, 5’-UUG CAU UUG GAC CAU GGU C-3’) or control RNA (50 nM) with a scrambled sequence. pCMV-myc mammalian expression vector was originally developed from Clontech, and general information for this vector is available at http://www.takara.co.kr/file/manual/pdf/PT3282-5.pdf. To determine the effect of osmotic stress on TonEBP and AVP expression, the cells were treated with NaCl (50 or 100 mM) for 24 h, which did not induce cell death.

### Real-Time PCR

To determine the effect of TonEBP on AVP expression during dehydration, bilateral punches of the PVN and SON were taken using 1.00 mm needles and referencing the *Stereotaxic Mouse Brain Atlas*. Samples were collected from mice that had been water-deprived for 2 days. Total RNA samples were extracted using the Sensi-TriJol™ reagent (Lugen Sci co., Ltd, Korea, Catalogue No. LGR-1117) according to the manufacturer’s instructions. cDNA was synthesized using MMLV reverse transcriptase (Beams Bio., Korea, Catalogue No. #3201). After reverse transcription, real-time PCR analyses were performed with Bright-Green 2X qPCR MasterMix-ROX (abm, Vancouver, Canada) using a StepOnePlus Real-Time PCR System (Applied Biosystems, Thermo Fisher Scientific, Waltham, MA, USA) for ∼40 cycles. The target DNA species were amplified by real-time PCR using the following primer sets: TonEBP sense primer, 5’-TGA CCC TTG AAC CAA CTG GA-3’; TonEBP antisense primer, 5’-GGA GCA GAA CCA GTC AA-3’; AVP sense primer, 5’-GCT TGT TTC CTG AGC CTG CT-3’; AVP antisense primer, 5’-GCT CCA TGT CAG AGA TGG CC-3’; β-actin sense primer, 5’-TGG AAT CCT GTG GCA TCC ATG AAA C -3’; β-actin antisense primer, 5’-TAA AAC GCA GCT CAG TAA CAG TCC G -3’. Data were normalized for gene expression using β-actin as an internal control, as an expression pattern of β-actin in our experimental sets did not differ between groups (data not shown). The 2^_ΔΔCT^ method (46) was used to analyze the relative quantification of gene expression.

### Promoter Assay

To determine whether TonEBP regulates AVP transcription, mHypoA cells were co-transfected with an AVP promoter-luciferase reporter vector and a TonEBP expression vector (300 ng) using jetPRIME**^®^** (Polyplus). The 5’ flanking region of the rat AVP gene (-2059 to +6 bp) that we used for this promoter analysis was cloned by PCR from rat genomic DNA based on sequence information deposited in the GenBank™ database (accession number: AF112362.1). The 2,065-nucleotide-long PCR product was inserted into a luciferase reporter plasmid (pGL2-promoter vector, Promega, Madison, WI, USA), and its sequence was confirmed by DNA sequencing. This 5’ flanking sequence of the rat AVP gene includes 4 conserved core domains (TGGAAANNYNY, N = any nucleotide, Y = pyrimidine nucleotide) for the binding of TonEBP (44). Transfection efficiency was normalized by co-transfecting a β-galactosidase reporter plasmid (pCMV-β-gal; Clontech, Palo Alto, CA) at 100 ng/well. Transfected cells were harvested 24 h after transfection, and luciferase activity was measured using a luciferase reporter assay system (Promega) according to the manufacturer’s protocols.

### Chromatin Immunoprecipitation (ChIP) Assays

mHypoA 2/28 cells were transiently transfected using jetPRIME**^®^** reagent with expression vector pCMV-myc containing the TonEBP coding region or control pCMV-myc. Independently, hypothalamic fragments were also collected from mice subjected to dehydration for 2 days. The detailed procedures and materials for the ChIP assays in this study followed a previous report (47). Briefly, both samples were lysed in 0.3 ml of cell lysis buffer (5 mM PIPES (pH 8.0), 85 mM KCl, 0.5% NP40 and protease inhibitors) for 10 min on ice, and nuclei were extracted and resuspended with nuclear lysis buffer. Chromatin was sheared by sonication and diluted 5-fold in ChIP dilution buffer. The reactions were incubated with 5 μg of rabbit anti-TonEBP antibody (Thermo Scientific, Waltham, MA, USA, Catalogue No. PA1-023) or normal rabbit IgG (Santa Cruz, Catalogue No. sc-2004) at 4°C overnight. Immune complexes were collected by reaction with 60 μl of salmon sperm DNA/protein A agarose and then washed with washing buffer that contained different concentrations of salts (150 mM–500 mM) and 0.25 M LiCl. DNA from the protein–DNA cross-links was extracted by incubating the reactions with extraction solution, and it was further purified with phenol/chloroform. PCR amplification was performed using 30 cycles of 94°C for 30 sec, 53°C for 30 sec, and 72°C for 30 sec, preceded by 94°C for 30 sec and followed by 72°C for 10 min using the following primer sets: TonEBP at -1930, forward, 5’-CCA TTC ATG AAG CAG CAC AG-3’; TonEBP at -1930, reverse, 5’-GGA AAC AGC TTC CTT GGT CA-3’; TonEBP at -1890, forward, 5’-GTT TCT GAC CAA GGA AGC-3’; TonEBP at -1890, reverse, 5’-CAG CAT AAA GTA GTG AAG GGC-3’; TonEBP at -330/-343, forward, 5’-CTG CTC CAA ACG TGC CAG-3’; TonEBP at -330/-343, reverse, 5’- GCC AGC CAT CAG GCT GT-3’.

### Western Blotting Analysis

Cytoplasmic as well as nucleus-specific proteins in the PVN and SON were isolated by using the Nucleus and Cytoplasmic extraction kit (Thermo Scientific, Catalogue No. 78833), according to the manufacturer’s instruction. Briefly, protein concentration was measured by the Bradford dye-binding assay (Bio-Rad, Hercules, CA, USA, Catalog No. 5000006), and then, equal amount of proteins from each sample were separated by SDS-PAGE, transferred onto the PVDF membranes by electrophoretic transfer. The membrane was blocked with 5% non-fat skim milk in TBS-tween, and then incubated with anti-TonEBP (Santa Cruz, Catalog No. sc-398171; 1:1000 dilution). The membrane was incubated with HRP-conjugated mouse secondary antibody (Cell signaling, Danver, MA, USA, Catalog No. 7076; 1:3000 dilution), and the immunoreactive signals were detected by Chemiluminescent detection reagent (Thermo Scientific, Catalog No. 34095). Protein density was normalized using an anti-Hsc70 antibody (Rockland, Limerick, PA, USA, Catalog No. 200-301-A28; 1:1000 dilution), and ImageJ software was utilized to analyze data.

### Statistical Analyses

Statistical analyses were performed in GraphPad Prism 9 software (GraphPad Software, San Diego, CA, USA). All data are expressed as the mean ± SEM. Data were analyzed with a one-way or two-way analysis of variance followed by the Tukey’s multiple comparison test for unequal replications. A Student’s t test was used to compare the two groups.

## Results

### Enhanced TonEBP Expression Within Hypothalamic AVP Neurons During Hyperosmolality

**Figure 1:**
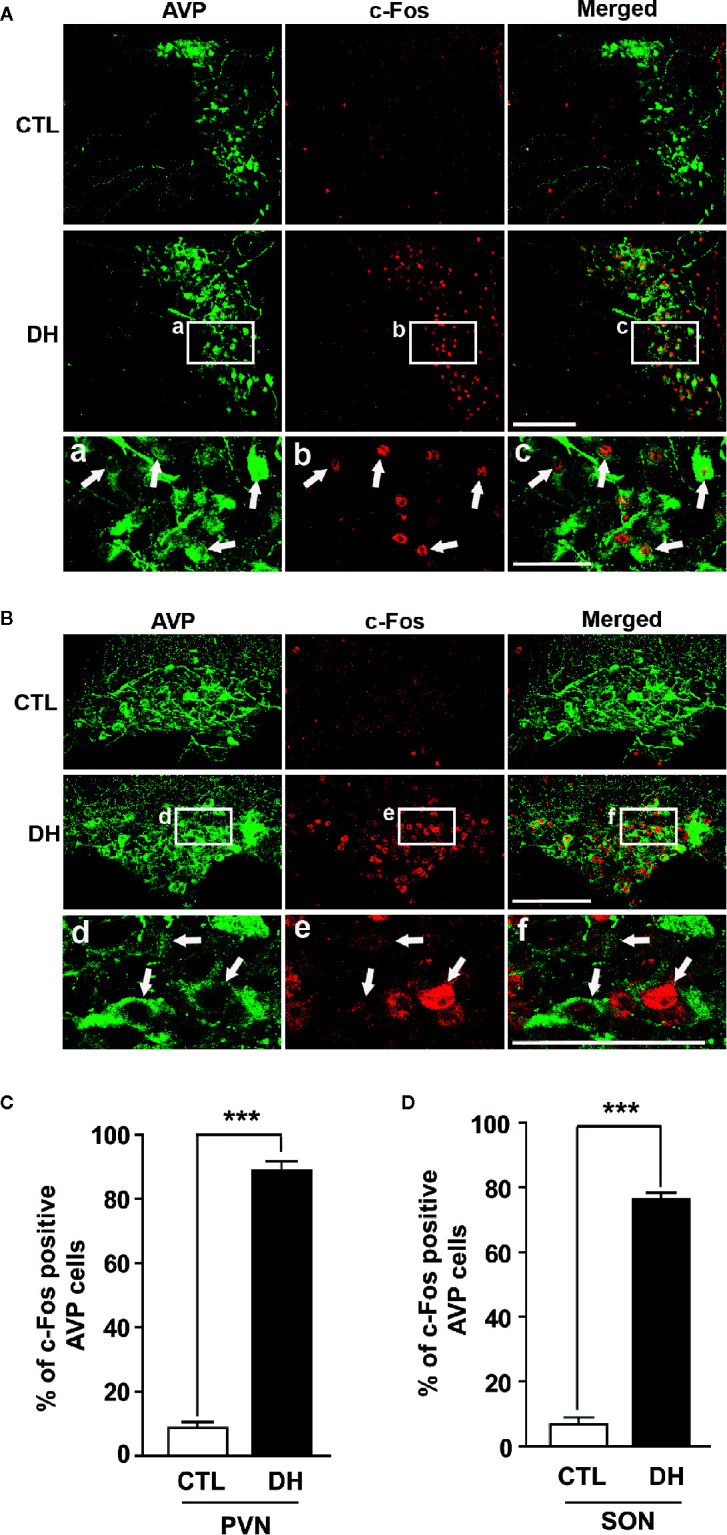
Dehydration-induced elevation of AVP cellular activity in the PVN and SON. Double immunohistochemistry was used to quantify the activity of AVP cells in the PVN and SON in mice deprived of water for 2 days and euhydrated control mice. **(A, B)** Representative images show overall AVP-immunoreactive cells (green) and c-Fos-immunopositive (red) AVP cells in the PVN **(A)** and SON **(B)**. High magnified images from water deprived condition clearly indicate nuclear localization of c-Fos immunoreactivity in the AVP cells with arrows in both regions. **(C, D)** Quantificational analyses clearly demonstrate that dehydration resulted in an increase in c-Fos immunoreactivity within the AVP cells in both regions. CTL, euhydrated control; DH, 2 days of dehydration. N= 3 sections, each with 4 mice. Data are presented as mean ± SEM. ***p < 0.001. Scale bar = 100 μm.

**Figure 2:**
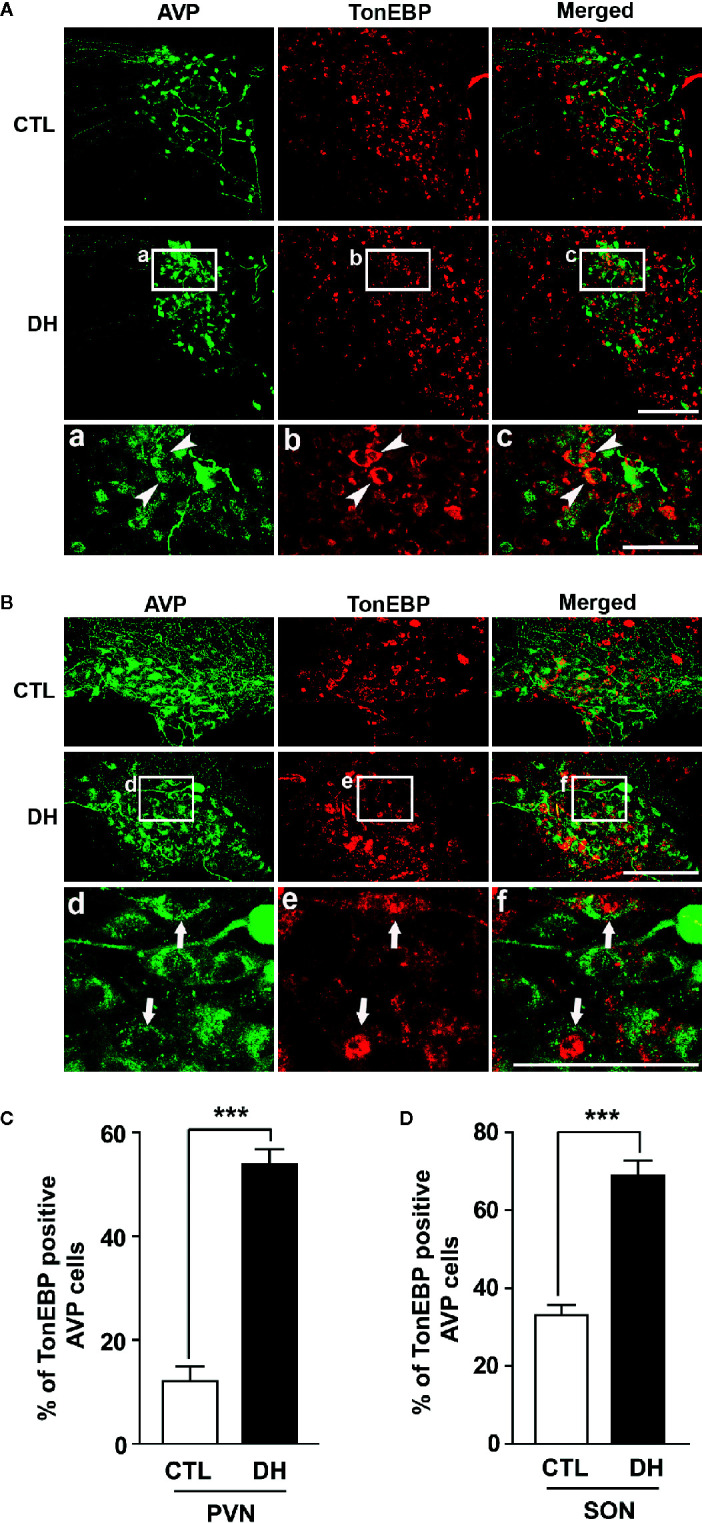
Dehydration-induced elevation in TonEBP expression in AVP cells in the PVN and SON. Double immunohistochemistry was used to quantify the expression of TonEBP in AVP cells in the PVN and SON in mice deprived of water for 2 days and euhydrated control mice. **(A, B)** Representative images show TonEBP-immunoreactive (red) and AVP-immunoreactive cells (green) in the PVN **(A)** and SON **(B)**. Interestingly, high magnified images from dehydration condition indicate TonEBP nuclear translocation in the SON (arrows), but not in the PVN (arrow heads). **(C, D)** Quantificational analyses clearly demonstrate that dehydration resulted in an increase in TonEBP immunoreactivity in the AVP cells in both regions. CTL, euhydrated control; DH, 2 days of dehydration. N= 3 sections, each with 4 mice. Data are presented as mean ± SEM. ***p < 0.001. Scale bar = 100 μm.

**Figure 3:**
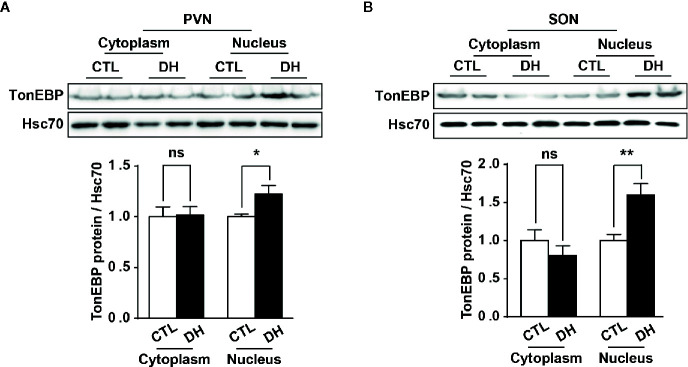
Dehydration-induced nuclear translocation of TonEBP in the PVN and SON. Western blotting analysis was used to quantify cytoplasmic or nucleus TonEBP in the PVN and SON in mice deprived of water for 2 days and euhydrated control mice. Representative images show intracellular region-specific TonEBP proteins in the PVN **(A)** and SON **(B)**, and quantificational analyses demonstrate that dehydration resulted in an increased TonEBP nuclear translocation in both regions. CTL, euhydrated control; DH, 2 days of dehydration; N= 5 mice per group. Data are presented as mean ± SEM. *p < 0.05; **p < 0.01; ns, not significant.

The activity of hypothalamic AVP cells, measured using c-Fos immunoreactivity as a molecular marker for cellular activity, is elevated under hyperosmolality conditions in multiple animal models, along with increased levels of AVP gene and protein expression (31, 48, 49). To confirm that observation in our *in vivo* animal model, we induced hyperosmolality with 2 days of water deprivation and measured c-Fos immunoreactivity in AVP cells in the PVN and SON (**Figure 1**). As expected, the activity of AVP cells in both regions was enhanced by water-deprived hyperosmolality compared with euhydrated control mice (average percentage of c-Fos positive AVP cells in the PVN: 88.9% vs. 8.6%, and in the SON: 78.8% vs. 8.6%, in hyperosmolality vs. control, respectively). Furthermore, double IHC was performed to determine whether hyperosmolality-dependent production of TonEBP occurs in hypothalamic AVP cells (**Figure 2**). In line with the findings from studies in chickens, TonEBP immunoreactivity in AVP cells in the PVN and SON was elevated in mice in the hyperosmolality condition compared with the euosmotic control animals (average percentage of TonEBP positive AVP cells in the PVN: 54.0% vs. 13.8%, and in the SON: 68.9% vs. 33.1%, in hyperosmolality vs. control, respectively). Interestingly, the intracellular distribution of TonEBP seemed to differ between the PVN and the SON. It seemed like that TonEBP immunoreactivity was abundantly accumulated in the cytoplasm of PVN AVP cells, while majority of AVP cells in the SON showed a translocation of TonEBP immunoreactivity to the nucleus. However, it was unable for us to quantify cytoplasmic- versus nucleus-specific TonEBP in AVP cells by our immunofluorescence. Therefore, we extracted cytoplasmic- or nucleus-specific proteins from the PVN and SON, and performed western blotting analysis to quantify intracellular subregion-dependent TonEBP proteins following 2 days of dehydration (**Figure 3**). Our results indicated a hyperosmolality-induced increased TonEBP translocation from the cytoplasm to the nucleus, both in the PVN and SON. Although indirect, these results support a possibility of increased intracellular TonEBP translocation to the nucleus of AVP cells in both hypothalamic regions following dehydration.

### TonEBP as a Transcriptional Activator of AVP Gene Expression

**Figure 4:**
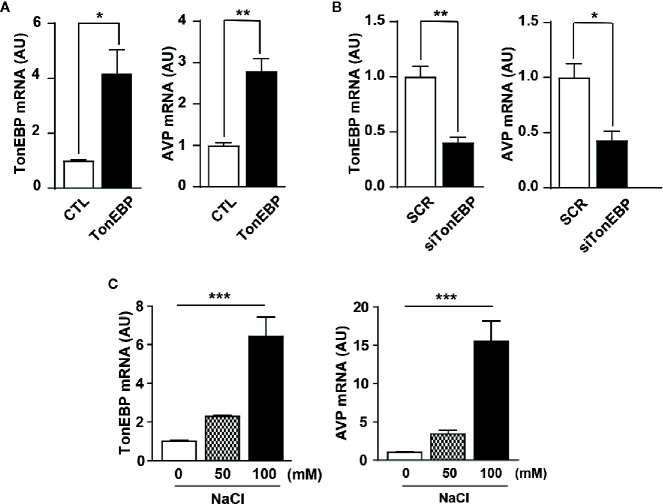
Role of TonEBP in the activation of AVP gene transcription. In *in vitro* experiments, mouse hypothalamic (mHypoA) cells were subjected to real-time PCR to determine interactions between TonEBP and AVP gene transcription. **(A)** The mHypoA cells were transfected with either an expression vector carrying the TonEBP-coding region (500 ng per well) or a control vector for 24 h, and the mRNA expression of TonEBP and AVP was quantified. **(B)** Independently, TonEBP siRNA (siTonEBP, 50 nM per well) or a scrambled control sequence of siRNA (SCR) was transfected into mHypoA cells for 24 h, and the mRNA expression of TonEBP and AVP was quantified. **(C)** Gene transcription of TonEBP and AVP in an environment of osmotic stress was determined in mHypoA cells exposed to different doses of NaCl for 24 h. GAPDH was used as an internal control for all experiments. All data are presented as means ± SEM (n = 6). AU, arbitrary units. *p < 0.05; **p < 0.01; ***p < 0.001.

Next, we used *in vitro* experiments with mHypoA cells to examine the role of TonEBP in AVP gene transcription. The mHypoA cells were transfected with either a TonEBP expression vector or TonEBP siRNA to produce gain-of-function and loss-of-function of TonEBP, respectively, under normal osmotic conditions. A real-time PCR analysis measured the level of gene transcription of both TonEBP and AVP (**Figures 4A, B**). Importantly, the patterns of gene expression for TonEBP and AVP were synchronized with each other, with AVP gene transcription up- or down-regulated as the level of TonEBP moved up or down, respectively (419% in TonEBP mRNA and 280% in AVP mRNA with TonEBP overexpression; 47% in TonEBP mRNA and 43% in AVP mRNA with TonEBP siRNA). Using the same cell line, the gene expression profiles of both TonEBP and AVP were determined in different extracellular hyperosmolality conditions. To induce different degrees of extracellular hyperosmolality, different doses of NaCl were applied to the cell culture medium. As shown in **Figure 4C**, the gene expression of both molecules increased gradually following an increase in osmolality (TonEBP mRNA, 229% and 641% of control with 50 mM and 100 mM NaCl treatment, respectively; AVP mRNA, 344% and 1554% of control with 50 mM and 100 mM NaCl treatment, respectively). These results indicate that TonEBP could act as a transcriptional activator of the AVP gene in a high osmolality environment.

### TonEBP Directly Regulates Hyperosmolality-Dependent AVP Gene Expression

**Figure 5:**
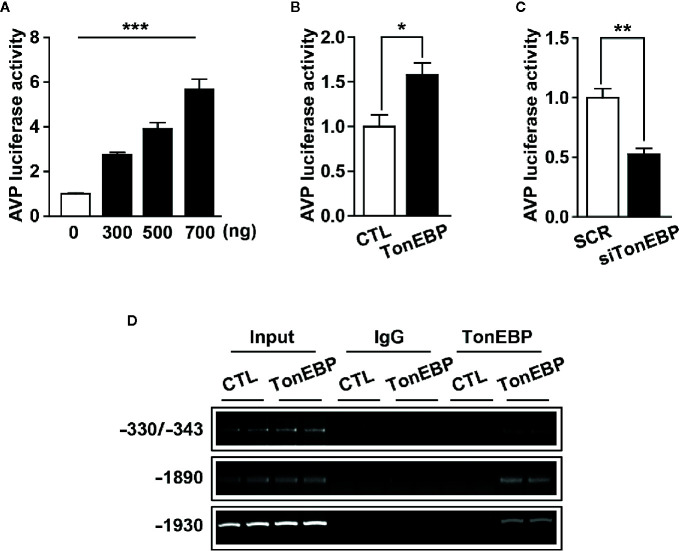
TonEBP binds directly to AVP promoter region and activates dehydration-induced AVP gene transcription. **(A)** Luciferase assays show an increased promoter activity in parallel with an increasing number of AVP-promoter-luciferase constructs. **(B, C)** Luciferase-reporter vectors containing the 5’-flanking region of the AVP gene were co-transfected into mHypoA cells with 500 ng of TonEBP expression vector **(B)**, or 50 nM of TonEBP siRNA/control scrambled sequence **(C)** for 24 h. Then, beta-galactosidase assays were performed to quantify TonEBP-dependent AVP promoter activity. **(D)** ChIP assays were performed to verify whether TonEBP can directly bind to the AVP promoter. Mouse hypothalamic (mHypoA) cells were transiently transfected with pCMV-myc expression vector containing the TonEBP coding region (TonEBP) or control pCMV-myc (CTL), and nuclear DNA samples were immunoprecipitated with TonEBP antibody. Then, PCR amplification was performed using primer sets targeting the indicated TonEBP binding motifs on the AVP gene. Normal rabbit IgG was used as a negative control. All data are presented as means ± SEM (n = 6). **p < 0.01; ***p < 0.001.

Our results clearly demonstrate a positive correlation between TonEBP and AVP expression in response to osmotic stress. The 5’-flanking promoter region of the AVP gene is highly conserved between species, and includes multiple potential TonEBP binding sites as characterized previously (35, 50) (**Supplementary Figures 1** and **2**), so we investigated whether TonEBP can bind directly to the promoter regions of the AVP gene to activate AVP gene transcription. First, we measured AVP promoter activity in response to different levels of TonEBP by analyzing the luciferase activity of the AVP promoter *in vitro*. The activity of the AVP promoter increased in parallel with an increasing amount of transfected promoter-luciferase plasmid, indicating that the AVP promoter was functional in our cell culture conditions (**Figure 5A**). TonEBP expression vectors induced an increase in AVP promoter activity (157% increase over the control) (**Figure 5B**). On the other hand, a TonEBP siRNA–mediated blockade of endogenous TonEBP synthesis lowered the AVP promoter activity (52% of control) (**Figure 5C**). Next, we investigated direct interaction between TonEBP and the AVP promoter region from *in vitro* and *ex vivo* systems using ChIP assay technology (**Figure 5D** and **Supplementary Figure 3**). For ex vivo studies, whereas the control mice had *ad libitum* access to water, the experimental mice underwent 2 days of water deprivation to induce dehydration. Hypothalamic nuclear protein was extracted and precipitated by the TonEBP antibody, and then a set of PCR primers was used to amplify the endogenous binding motifs of the AVP promoter to TonEBP for PCR analysis. Our results demonstrate that TonEBP binds directly to the AVP promoter regions at -1930 and -1890 and between -330 and -343 and that the binding activity was elevated during dehydration (**Supplementary Figure 3**). These findings were further confirmed in *in vitro* system using the mHypoA 2/28 cell line with the expression vector containing the TonEBP coding region, and the results were similar as described above in *ex vivo* experiments (**Figure 5D**). These results indicate that enhanced binding between TonEBP and the AVP gene promoter directly activates dehydration-dependent AVP gene transcription.

**Figure 6:**
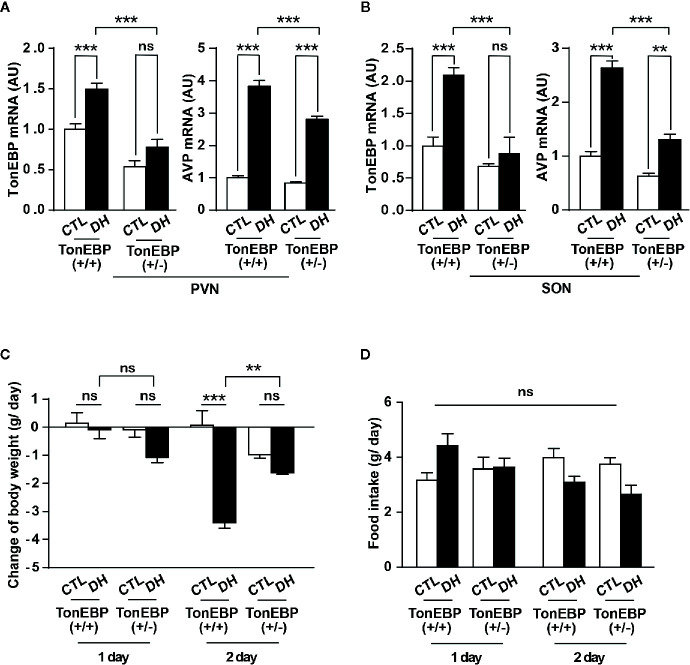
*In vivo* verification of the effects of TonEBP on dehydration-dependent AVP gene transcription and metabolic adaptation. **(A, B)** TonEBP haploinsufficiency [TonEBP (+/-)] and control [TonEBP (+/+)] mice underwent either 2 days of dehydration (DH) or euhydration (CTL), and gene transcription of TonEBP/AVP in the PVN **(A)** and SON **(B)** was quantified by real-time PCR. AU=arbitrary units. **(C, D)** Daily body weight gain **(C)** and daily food intake **(D)** from the same experimental animals were also measured. All data are presented as means ± SEM (n = 4). **p < 0.01; ***p < 0.001; ns, not significant.

To confirm our findings *in vivo*, we adapted a mouse model with TonEBP haploinsufficiency and measured the mRNA levels of TonEBP and AVP in the PVN and SON under dehydration (**Figures 6A, B**). The use of TonEBP haploinsufficiency (+/-) mice is necessary because homozygous TonEBP knockout (-/-) is lethal to animals during development (39). In concert with other results (31), dehydration for 2 days produced a significant increase in mRNA levels of TonEBP in the hypothalamic target regions of the TonEBP (+/+) mice (TonEBP mRNA, 149% of control level in PVN and 210% in SON), but not of the TonEBP (+/-) mice (**Figures 6A, B**), indicating that the entire TonEBP genetic components are necessary in these brain regions for the dehydration-dependent full expression of TonEBP. Importantly, our results further demonstrated a dehydration-induced increase in AVP mRNA levels in the target regions of both animal groups (AVP mRNA from TonEBP (+/+) mice, 382% of control level in PVN and 263% in SON; AVP mRNA from TonEBP (+/-), 332% of control level in PVN and 208% in SON), although a degree of elevation was significantly reduced in TonEBP (+/-) animals (AVP mRNA, 27% reduction to the dehydrated TonEBP (+/+) mice in PVN and 50% reduction in SON). These results suggest an involvement of other mechanisms, together with TonEBP, in the dehydration-dependent AVP gene expression in the PVN and SON. In addition, we investigated a dehydration-induced anorectic effect in TonEBP (+/-) mice (**Figures 6C, D**). Previous investigations, including by our group, have demonstrated that metabolic parameters between TonEBP (+/+) and TonEBP (+/-) mice, such as food intake, body weight gain, glucose and insulin metabolism, and physical activity, are comparable to each other under normal diet conditions (41, 44). However, in this study we found that acute dehydration reduced the daily body-weight gain in the TonEBP (+/+) mice, but not in the TonEBP (+/-) mice [on day 2 of dehydration, -3.4 vs. 0.1 g/day in TonEBP (+/+) mice; -1.6 vs. -1.0 g/day in TonEBP (+/-) mice, dehydrated vs. control, respectively]. Comparing with the daily body-weight changes, the dehydration-dependent daily change in food intake was comparable between the two groups [on day 2 of dehydration, 3.1 vs. 4.0 g/day in TonEBP (+/+) mice; 2.7 vs. 3.8 g/day in TonEBP (+/-) mice, dehydrated vs. control, respectively], although dehydration for 2 days in both animal models showed a considerably reduced food intake when compared to the euhydrated state. These results indicate that TonEBP-dependent hypothalamic AVP synthesis could affect dehydration-induced changes in metabolic parameter.

## Discussion

In the brain, AVP-producing cells are densely clustered within the hypothalamic PVN and SON, although scattered AVP cells are also observed in other hypothalamic regions (18). The concentration of AVP in both the brain and plasma depends on the production and release of AVP from those hypothalamic AVP cells. Hypothalamic AVP is strongly implicated in a wide range of physiological processes, including fluid and cardiometabolic homeostasis. For example, deficiency or overproduction of hypothalamic AVP leads to the development of diabetes insipidus or metabolic problems, including obesity, hypertension, and multiple forms of diabetes, respectively (4, 26, 51–53). Therefore, it is critical to tightly regulate the synthesis, release, and degradation of AVP. Although diverse physiological factors have been found to influence the release of AVP (19–21, 27, 29, 30), it remains unclear what intracellular molecular mechanisms within hypothalamic AVP cells regulate AVP synthesis. Hyperosmolality-sensitive AVP gene activation is of particularly great interest because plasma hyperosmolality is a powerful stimulator of AVP production (54).

In this work, we used *in vitro*, *in vivo*, and *ex vivo* experiments to demonstrate the role of TonEBP in dehydration-dependent hypothalamic AVP gene transcription in rodents. Our results demonstrate synchronized patterns of TonEBP and AVP gene expression in response to osmotic stress. We further found that TonEBP can bind directly to the promoter region of AVP and that TonEBP is responsible for, at least in some degree, dehydration-induced AVP gene expression in the hypothalamic PVN and SON in rodents. This TonEBP-mediated hypothalamic AVP gene expression is necessary for dehydration-dependent body weight loss to occur.

In the rodent brain, TonEBP is widely distributed in neuronal and non-neuronal populations, however, tonicity-dependent induction of TonEBP is observed exclusively in neuronal cells (37). Extracellular hypertonic status induces gene transcription of TonEBP in the hypothalamic PVN and SON (36). Once produced, TonEBP can be rapidly translocated to the nucleus, similar to other transcription factors, to activate the transcription of genes for osmoprotective molecules and protect the cells from osmotic stress (32, 55). In line with other observations (36), our immunohistochemistry results clearly demonstrate an overall dehydration-induced elevation in TonEBP protein in the PVN and SON, especially in the AVP cells in those regions (**Figure 2**). In addition, our results clearly demonstrate an increased TonEBP nuclear translocation in SON AVP cells during dehydration by the localization of TonEBP immunoreactivity (**Figure 2**), western blotting analysis (**Figure 3**), and the results of our ChIP assays (**Figure 5D** and **Supplementary Figure 3**). However, the results of dehydration-induced nuclear translocation of TonEBP in the PVN AVP cells differed from the SON; while abundant TonEBP immunoreactivity following dehydration is observed from our immunohistochemistry, it seemed mostly to be localized in the cytoplasm of AVP cells in this brain region (**Figure 2**). However, our western blotting analysis indicate dehydration-induced increase in TonEBP nuclear translocation in the PVN as well (**Figure 3**). Therefore, detailed investigation that points to PVN AVP-specific and dehydration-dependent TonEBP mechanism is further necessary.

It is worth noting that TonEBP responds to changes in the extracellular osmotic environment and regulates tissue- and cellular-phenotype-specific gene expression for intracellular and matrix homeostasis (32, 55, 56). Therefore, TonEBP has appeared to be a local regulator of fluid balance, rather than a global player. However, our results suggest that hypothalamic TonEBP could act as a global regulator of fluid homeostasis *via* AVP cells in the PVN and SON. Within the hypothalamus, dramatic inter- and intra-cellular modifications occur at the genetic and molecular levels in response to changes in local extracellular osmolality, in concert with changes in plasma volume and osmolality (57, 58). Therefore, the local hypothalamic extracellular osmotic profile is, to a certain degree, a mirror image of the global plasma fluid parameters. This phenomenon could be largely mediated by the forebrain sensory CVOs, including the SFO and OVLT, because those nuclei include osmosensitive cells that directly connect with the circulation and establish massive synaptic networks to the PVN and SON (9, 11, 13, 15, 59). Altogether, these results suggest that the level of TonEBP in the PVN and SON represents not only the local extracellular osmotic status, but also the plasma osmotic environment, and that TonEBP in these regions may be a global regulator of water physiology through the AVP system.

Clearly, TonEBP is the upstream transcription factor for hypothalamic AVP gene transcription, and it contributes to hyperosmolality-dependent AVP synthesis. As a next step, we used a TonEBP haploinsufficiency mouse model to investigate the physiological function of the hypothalamic TonEBP–AVP system in dehydration-dependent metabolic adaptation. TonEBP knockout animals die during development, so TonEBP haploinsufficiency mice have been used extensively to study the physiological and behavioral functions of TonEBP *in vivo* (39, 41, 44). While mRNA levels of TonEBP in the PVN/SON of control mice are increased in response to dehydration, this phenomenon is attenuated in TonEBP haploinsufficiency animals, indicating that TonEBP gene expression in response to dehydration is impaired in the animals with TonEBP haploinsufficiency. However, AVP gene expression in the PVN/SON is elevated in both animal groups, although a degree of increased AVP gene expression is significantly reduced in TonEBP haploinsufficiency animals. These results suggest that TonEBP may be necessary, but not sufficient by itself, for dehydration-dependent AVP gene expression in the PVN/SON. This also suggests an involvement of other signaling(s), in addition to TonEBP, in such a case. In support of this view, recent investigations have demonstrated that cAMP responsive element-binding protein-3 like-1 (CREB3L1) and caprin-2 are distributed in the PVN/SON, possess an ability to bind directly to promoter and mRNA regions of AVP, respectively, and play a role in AVP-mediated and osmotic stress-dependent physiological adaptations (60, 61). Therefore, it is reasonable to note that a cellular and molecular signaling complex, including TonEBP, is contributing to osmotic stress-dependent hypothalamic AVP synthesis.

Generally, dehydration leads to an increase in hypothalamic AVP cellular activity and plasma AVP concentrations (19–21, 62) and to a decrease in food intake and subsequent body-weight gain in rodents to preserve the body’s water and metabolic homeostasis (63–65). However, we found that the dehydration-dependent reduction in body weight in TonEBP (+/+) animals was attenuated in TonEBP haploinsufficiency mice, with a reduced AVP expression in the PVN/SON. It is noteworthy that although our results for food intake did not meet a statistical significance, they showed a considerable reduction in food intake following dehydration in both animal groups. These results suggest a possible involvement of the TonEBP-AVP system in the PVN/SON in osmotic stress-mediated metabolic adaptation, although detailed investigation to uncover a physiological role of the hypothalamic TonEBP-AVP system in stress-dependent metabolic adaptation is further necessary. The anatomical and molecular characteristics of hypothalamic AVP cells are highly complex. For example, multiple investigations have suggested that PVN AVP cells establish multiple synaptic networks, not only to the neurohypophysis but also to several extra-neurohypophyseal targets in the brain (52, 66, 67). These extra-neurohypophyseal projections of PVN AVP cells have been suggested to be implicated in anxiety and stress responses (66, 67). In addition, polysynaptic networks between PVN AVP cells and adipose tissue have also been characterized and suggested to play a role in regulating adipose metabolism (68). Unlike the PVN, most AVP cells within the SON project their fibers to the neurohypophysis to regulate plasma AVP levels (69). Independent investigations have also demonstrated a complex profile of metabolic receptors within hypothalamic AVP cells (27, 70–73). Therefore, hypothalamic AVP–driven metabolic outcomes, such as food intake, body weight gain, energy expenditure, and adipose metabolism, could be mediated by different combinations of anatomical and molecular profiles in individual AVP cells. Therefore, alterations in body weight gain independent of feeding behavior, if this is the case, could possibly be driven by modifications in energy expenditure. In any case, TonEBP contributes to, at least in part, hyperosmolality-dependent AVP synthesis in the hypothalamic neuroendocrine nuclei, such as the PVN and SON.

## Data Availability Statement

The raw data supporting the conclusions of this article will be made available by the authors, without undue reservation.

## Ethics Statement

The animal study was reviewed and approved by University of Ulsan for the Care and Use of Laboratory Animals (permission number, BJL-20-050).

## Author Contributions

DK, JJ, and BL designed the experiments, interpreted results, and wrote the manuscript. DK and KK performed and analyzed most of the experiments. TL and JK performed the histological analyses. HE performed the real-time PCR analysis. JP provided intellectual input. All authors contributed to the article and approved the submitted version.

## Funding

This research was supported by the Priority Research Centers Program (2014R1A6A1030318) and research funds through the National Research Foundation of Korea (NRF-2020R1I1A1A01068381).

## Conflict of Interest

The authors declare that the research was conducted in the absence of any commercial or financial relationships that could be construed as a potential conflict of interest.

## Supplementary Material

Supplementary Figure 1 Putative AVP 5’-franking promoter sequences in rats. The sequences are obtained from the NCBI GenBank™ database; accession number AF112362.1, and nucleotides are numbered by assigning position +1 to the ATG translation start site. Sequences with underline represent putative TonEBP binding domains as indicated in the Method.

Supplementary Figure 2 Putative AVP 5’-franking promoter sequences from mouse, rat and human are obtained from the NCBI GenBank™ database; accession gene ID 11998 for mouse, 24221 for rat, and 551 for human, and sequences are aligned, analyzed for the conserved TonEBP binding motifs among species, and deposited at online http://www.ebi.ac.uk/Tools/services/web/toolresult.ebi?tool=clustalo&jobId=clustalo-E20210119-084011-0197-37166704-p1m. As indicated with red boxes, putative TonEBP binding motifs are highly conserved between mouse and rat, and with a less degree with human.

Supplementary Figure 3 ChIP assays were performed to verify whether TonEBP can directly bind to the AVP promoter under dehydration. Hypothalamic nuclear DNA samples from animals dehydrated for 2 days (DH) and euhydrated control animals (CTL) were immunoprecipitated with TonEBP antibody. Then, PCR amplification was performed using primer sets targeting the indicated TonEBP binding motifs on the AVP gene.
