# Supplementary figures and images for: Transcription Factor TonEBP Stimulates Hyperosmolality-Dependent Arginine Vasopressin Gene Expression in the Mouse Hypothalamus
Source: Front Endocrinol (Lausanne). 2021 Mar 16;12:627343. doi: 10.3389/fendo.2021.627343 (PMC8008816; doi:10.3389/fendo.2021.627343)

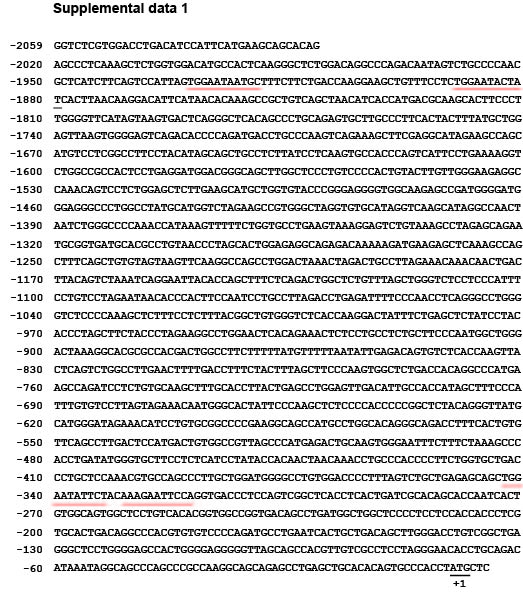

Supplement: Supplementary Figure 1 — Putative AVP 5’-franking promoter sequences in rats. The sequences are obtained from the NCBI GenBank™ database; accession number AF112362.1, and nucleotides are numbered by assigning position +1 to the ATG translation start site. Sequences with underline represent putative TonEBP binding domains as indicated in the Method. [file Image_1.jpg]

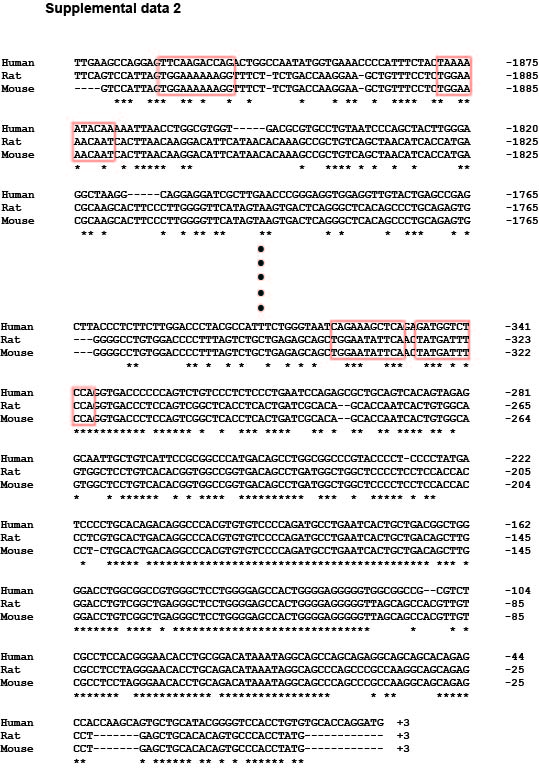

Supplement: Supplementary Figure 2 — Putative AVP 5’-franking promoter sequences from mouse, rat and human are obtained from the NCBI GenBank™ database; accession gene ID 11998 for mouse, 24221 for rat, and 551 for human, and sequences are aligned, analyzed for the conserved TonEBP binding motifs among species, and deposited at online http://www.ebi.ac.uk/Tools/services/web/toolresult.ebi?tool=clustalo&jobId=clustalo-E20210119-084011-0197-37166704-p1m. As indicated with red boxes, putative TonEBP binding motifs are highly conserved between mouse and rat, and with a less degree with human. [file Image_2.jpg]

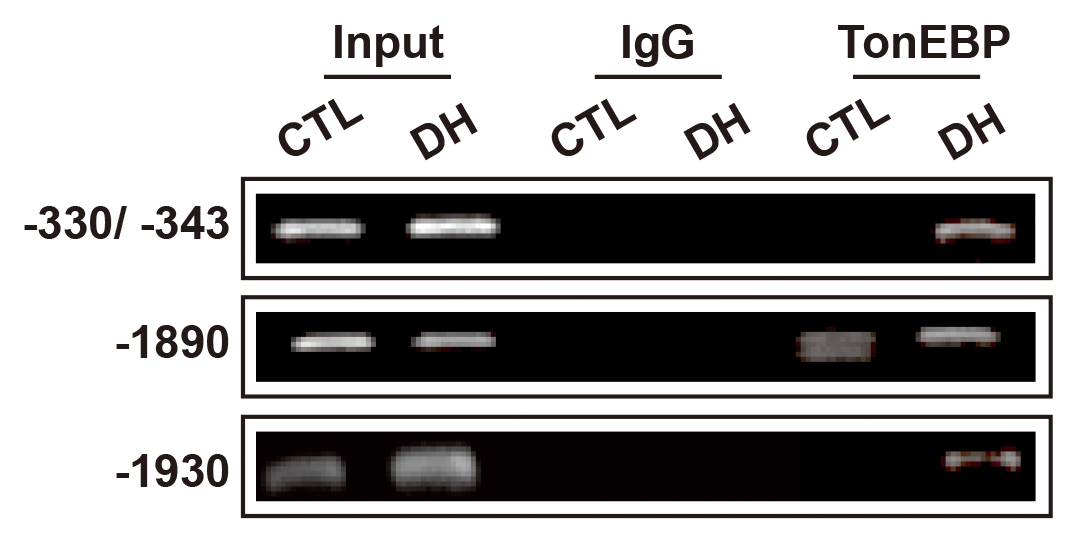

Supplement: Supplementary Figure 3 — ChIP assays were performed to verify whether TonEBP can directly bind to the AVP promoter under dehydration. Hypothalamic nuclear DNA samples from animals dehydrated for 2 days (DH) and euhydrated control animals (CTL) were immunoprecipitated with TonEBP antibody. Then, PCR amplification was performed using primer sets targeting the indicated TonEBP binding motifs on the AVP gene. [file Image_3.tif]
